# Profile of acute poisoning in three health districts of Botswana

**DOI:** 10.4102/phcfm.v1i1.10

**Published:** 2009-05-05

**Authors:** Mary Kasule, Ntambwe Malangu

**Affiliations:** 1 Department of Natural Sciences, Institute of Health Sciences, Botswana; 2 Department of Epidemiology, University of Limpopo, South Africa

**Keywords:** acute poisoning, case fatality, outcome, Botswana, Gaborone

## Abstract

**Background:**

This study sought to characterise acute poisoning cases seen in three health districts of Botswana.

**Method:**

A retrospective review of patients’ records was conducted and included patients treated from January 2004 to December 2005. Data on the demographic status of the patients, information about the poisonous agent(s) involved, and the circumstances and outcomes of the poisoning incidents were recorded on a pre-tested data collection form.

**Results:**

A total of 590 cases of acute poisoning were included in the analysis. The most affected age category was that of children aged less than six years, who constituted 33.4% of the cases. Most incidents were recorded in the urban district of Gaborone. Seventy-eight percent (78%) of the incidents were accidental, with the remainder being intentional. The poisonous agents involved were pharmaceuticals (26.6%), natural toxins (25.6%), household products (14.6%), foods (14.4%), alcohol (6.9%), traditional medicines (4.7%), unspecified agents (3.2%), and agrochemicals (2.7%). The most common route of poison exposure was by oral (82.2%), followed by dermal contact (16.5%), while the inhalation of gases occurred in 1.2% of cases. An incidence rate of 4.7/1000, a case fatality rate of 3.8/100, and 1.5% of deaths were recorded over the two-year period.

**Conclusion:**

In conclusion, it can be stated that acute poisoning involved mainly young children and resulted in an incidence rate of 4.7/1000, a case fatality rate of 3.8/100, and 1.5% of deaths over the two-year period. There were differences based on age category, gender and residence of the victims, the types of toxic agents involved, as well as the circumstances and the outcomes of the poisoning incidents. Given the fact that pharmaceuticals, natural toxins, household products and foods were the agents most commonly involved, targeted interventions should take these differences into account in addressing the problem of acute poisoning.

## INTRODUCTION

Acute poisoning is an important cause of morbidity and mortality worldwide. It is estimated that poisoning contributes to more than 1 million illnesses and up to half a million deaths each year, yet it is preventable and treatable.^1^ Accidental poisoning occurs mainly among children, with about 1.2 million children younger than six years being poisoned globally. It is also well established that more male than female children are victims of poisoning. Moreover, deliberate poisoning and a higher fatality rate are more common among adults.^2^ However, studies concerning poisoning in Botswana have been scant, although it has been reported that poisoning contributes about 6.7% of the total injuries and is ranked third next to falls and automobile accidents among external causes of mortality.^3^ Yet for programming purposes, it is important to distinguish between intentional and accidental poisoning, as well as to identify the poisoning agents responsible for the incidents. The objectives of this study, therefore, were to determine the common toxic agents, to describe the characteristics of the victims, the circumstances and the outcomes of the incidents, and to compare outcomes with regard to the demographic variables and between districts.

## METHOD

This retrospective study was designed to provide descriptive data for all patients that attended the accident and emergency departments of four hospitals in three health districts of Botswana, namely Gaborone, Kgatleng and South-East, over a two-year period as a result of acute poisoning. The hospitals serve an estimated population of about 320 137 people (54.6% of the country's population), providing emergency facilities and hospital care, and cater for referrals from primary health care centres. Hospitals in the Gaborone district function as tertiary and referral hospitals, receiving patients from other districts hospitals as well as from private-sector facilities in Gaborone.

The accident and emergency (A&E) manual register in each of the four hospitals was used to identify cases that matched the case definition of poisoning according to the International Classification of Disease (ICD-10).^4^ Data regarding age, gender, residence, toxic agents, patient involvement, route of exposure and outcome were collected from the accident and emergency department registers as well as from the admission records. Pharmaceutical agents were classified into different groups based on their pharmacological classes. The outcome was assessed as to whether the patient was discharged alive or had died. Data gathered were coded for computer analysis using SPSS version 13.0 for Windows. Inferential statistics were used to draw conclusions about the studied population in the three health districts, and the incidence rate was calculated. The p value was set at < 0.05 for statistical significance, and the case fatality rate was calculated for each institution and summed for the whole sample.

## RESULTS

### Frequencies and rates

The frequencies and rates of poisoning were analysed first by district and then by age and gender. A total of 590 cases were recorded as being due to acute poisoning at the accident and emergency departments of the four hospitals in the three health districts over the two-year study period (January 2004 to December 2005). Out of a total of 590 cases, 499 (84.6%) were hospitalised. As shown in Table 1, the majority of poisoning cases were recorded (65.7%) in the Gaborone health district. The incidence rate was 4.7/1 000, nine (1.5%) deaths were recorded and the case fatality rate (CFR) was 3.8/100 over the two-year period. Of those who died, three were poisoned by pharmaceuticals, two by traditional medicines, two as a result of food, one by plants and one due to snake envenomation. Analysis by age category showed that two deaths occurred in each of the following age groups: over 30 years, 20 to 30 years, six to 12 years, and younger than six years. One death occurred in the 13- to 19-year age group. No statistically significant relationship was observed between age and outcome (χ 2 = 1.408, p = 0.843). Analysis by districts showed that eight (88.8%) fatalities occurred in Gaborone and one in South-East district.

**TABLE 1:** Incidence and case fatality rates of poisoning in the three districts

DISTRICT	TOTAL POPULATION	NO. OF CASES	PERCENTAGE (%)	TOTAL DEATHS	CASE FATALITY RATE/100	INCIDENCE RATE/1000
Gaborone	186,087	338	65.7	8	2.4	1.8
Kgatleng	73, 507	132	22.4	0	0	1.8
South-East	60,623	70	11.9	1	1.4	1.1

**TOTAL**	**320, 137**	**590**	**100**	**9**	**3.8**	**4.7**

**FIGURE 1:**
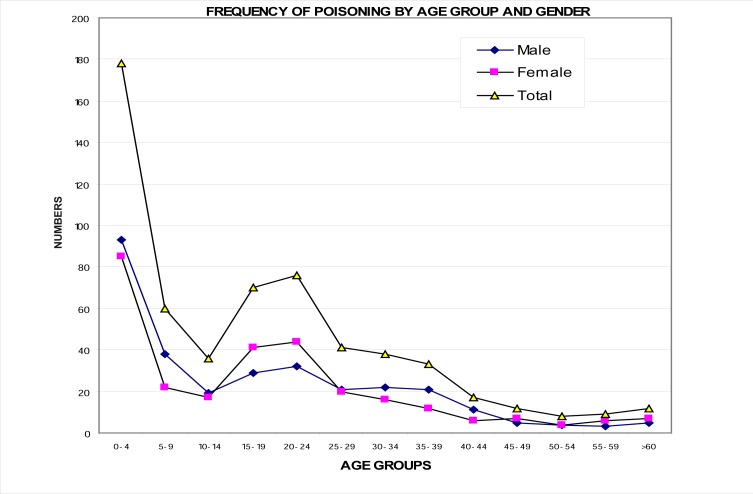
Frequency of poisoning by age group and gender in all three health districts

Further analysis revealed that the frequency for males (51.4%) was slightly higher than that for females (48.6%), and that the peak frequency occurred at the age of one to five years (197, 33.4%), and then in young adults aged 20 to 30 years (114, 19.3%). There was a drop in poisoning cases among the six- to 10-year age category (50; 8.5%) and those over 60 years of age (12; 2.0%), as shown in Figure 1.

The mean age of poisoned patients was 18.1 (+/- 16.1) years, ranging from 0.5 to 82 years. The majority of poisoning cases were accidental (88%), while the remainder (22%) were intentional especially in cases where patients were attempting to commit suicide. This was particularly the case for victims in the 20- to 30 years old age group. Regarding the place and time of incidence, most incidences occurred in urban areas (66.6%), and mostly at night (62.4%).

### Routes of exposure

The routes of exposure to poisonous agents were also investigated. Three common types of routes of exposure were recorded, namely ingestion, inhalation and injection via dermal contact. The most common route of exposure was via ingestion, constituting 489 cases (82.9%), followed by injection via dermal contact at 94 (31.6%). Only a few cases of poisoning by inhalation were recorded (7, 2.3%). The analysis by age category showed a significant association between age and route of exposure (χ^2^ = 41.942, *p*= 0.000), as more children younger than six years of age (184, 93.4%) and young adults aged 21 to 30 years (109; 84.5%) were poisoned via ingestion than any other age category. Dermal exposure affected mostly patients in the six- to 12-year-old category (33, 25.8%).

There were 94 (31.6%) cases of dermal contact due to insect stings and snake bites. This was the second most common route of exposure. Analysis by gender showed that more males (19.1%) than females (12.5%) were poisoned by this route.

### Poisonous agents

Table 2 shows the frequencies of categories of poisonous agents by district. Nine major categories of poisoning agents were recorded. The leading category was pharmaceuticals (157, 26.6%), followed by natural toxins (151, 25.6%), household products (86, 14.6%) and food toxins (85, 14.4%). Only a few cases of poisoning by alcohol (41, 6.9%), traditional medicines (28, 4.7%), agrochemicals (16, 2.7%), unspecified agents (19, 3.2%) and gases (1.2%) were reported. Analysis by district showed that Gaborone health district had more cases of pharmaceutical poisoning, followed by South-East and Kgatleng.

**TABLE 2:** Frequency of categories of poisoning agents and their distribution

TYPE OF POISON	GABORONE	KGATLENG	SOUTH-EAST	TOTAL	PERCENT
Alcohol	25	11	5	41	6.9
Agrochemicals	13	2	1	16	2.7
Carbon monoxide	6	1	0	7	1.9
Food toxins	45	8	32	85	14.4
Household	63	11	12	86	14.5
Natural toxins	78	20	53	151	25.6
Pharmaceuticals	126	12	19	157	26.6
Traditional medicine	23	1	4	28	4.7
Unspecified agent	9	4	6	19	3.2

**TOTAL**	**388**	**70**	**132**	**590**	**100**

In addition, more than half (53.6%) of the cases of poisoning by pharmaceuticals were recorded as ‘unknown drug’. Drugs that were specified as having been ingested intentionally or accidentally included paracetamol (12.3%), potassium permanganate (7.2%), Amytriptyline (3.6%) and analgesics (3.6%). Incidents involving poisoning by amoxicillin, Bromocriptine, ibuprofen, chlorpromazine, Phenobarbital, aspirin, cocaine and Imipramine were also recorded, although at a lower frequency ranging from 1.4 to 2.2%. Cases of multiple drug ingestion were also recorded. These cases involved alcohol and medicines being used together. Drug overdose was recorded mostly among young adults aged 20 to 30 years (38.9%) who used the drugs in attempted suicide, and in children younger than six years (24%), in which case it was considered accidental. Gaborone health district had the highest number of cases of poisoning by pharmaceuticals (81.8%), followed by South-East health district (11.5%), while Kgatleng health district had the lowest number of cases (6.5%).

With regard to household products, the leading poisoning agent was paraffin (63.5%), followed by household chemicals (11.7%). Paraffin ingestion was particularly common among the six- to 12-year-old group (77.9%), and most incidents reported were accidental. However, the use of paraffin in attempted suicide incidents was recorded in about 14.0% cases among young adults (20 to 30 years). Other household products recorded in low frequencies included acids, pesticides, petrol, engine cleaner, foam bath liquid soap, vinegar, hydrogen peroxide, methylated spirits, paints and soap powders. All cases of poisoning by household chemicals were recorded from the Gaborone health district. Regarding the circumstances of the poisoning, household products were ingested accidentally (83.7%), especially among children younger than six years of age.

**FIGURE 2:**
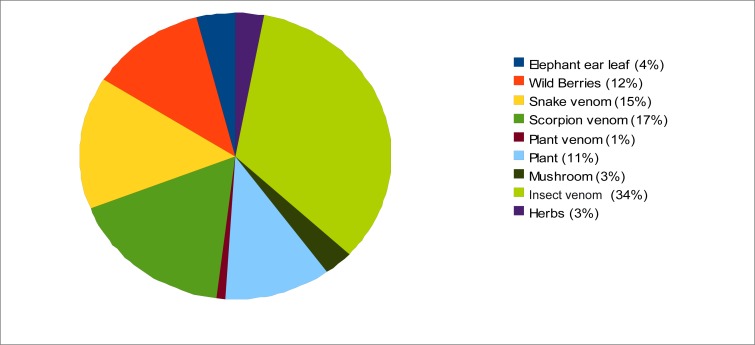
Distribution of natural toxic agents recorded in poisoning-related cases

Natural toxins contributed 25.6% of the total poisoning cases. These were mainly from plants, insects and animals (Figure 2). Plant poisons contributed 34% of the total cases of natural toxin poisoning, with wild berries accounting for 11.9%. The leaves of *Colocasia esculentum* (known locally as elephant ear), the only plant identified, were involved in 4.0% of cases. Another major contributor to natural toxin poisoning was insect stings (34%), in particular scorpion stings, while snake envenomation contributed 15% of cases. Analysis by age showed a significant association between age group and natural toxin poisoning (χ^2^ = 97.165, p = 0.000), as there were more children aged six to 12 years poisoned by natural toxins (30.5%) than any other age category. Arachnids, especially scorpions’ stings, were also recorded commonly as natural toxin poisoning agents for this age category. In patients aged 20 years and older, insect and snake bites were more common. Snake envenomation was particularly predominant among young adults aged 20 to 30 years and those older than 30 years. The length of stay for most patients that were admitted for natural toxin poisoning was reported to be an average of two days.

Other poisoning cases involved food poisoning, alcohol, traditional medicines and agrochemicals. Food poisoning predominantly involved children younger than five years of age (32.9%), as well as adults aged 30 years and older (27.1%), especially in the Gaborone health district (44.7%), followed by the South-East district (37.6%). All cases of food poisoning were accidental and more than 60% of them required hospitalisation. The length of stay in most cases of food poisoning was shorter than two days.

Regarding alcohol poisoning, there were 41 (6.9%) cases. The majority of them involved people in the age category of 20 to 30 years of age (45.5%). Males in this age category were affected more by alcohol poisoning than females, at a ratio of 2:1. Alcohol poisoning cases were intentional, with a few cases where the alcohol was mixed with drugs. The majority (75.6%) of patients with alcohol intoxication were hospitalised for fewer than two days.

Agrochemicals contributed 16 (2.7%) of the total poisoning cases, but the records did not specify the type of products that were ingested. Adults over 30 years of age (31.3%), and young adults 20 to 30 years of age (25.0%) were affected the most by poisoning with agrochemicals. In the incidents involving adults, the patients intended to commit suicide. Among children younger than six years of age, only four accidental cases were recorded. More males than females were poisoned by agrochemicals, with a male to female ratio of 1.6:1. Although all patients poisoned by agrochemicals were hospitalised, no fatalities were recorded and the average length of stay in hospital was less than two days.

Poisoning by traditional medicines was recorded in 28 (4.7%) of the poisoning cases. Analysis by age and gender showed that adolescents and adults were affected the most, making up about 28.6% of the total number of those poisoned by traditional medicines. In both these age categories, more males than females were affected, with a male to female ratio of 1:0.8. The majority of cases (92.9%) in which the patients were poisoned by traditional medicines required hospitalisation, and the average length of stay was shorter than two days.

Poisoning by gas inhalation was recorded in seven (1.1%) patients, and involved carbon monoxide. Four of the people affected were males, four were younger than 12 years old, and three were 20 years or older. Finally, some 19 (3.2%) cases of poisoning by unspecified poisonous agents were recorded. The majority of those affected were in the six- to 19-year age group. Eight patients were reported to have used these unspecified agents in an attempt to commit suicide, and six of them were adolescents aged 13 to 19. Five children younger than six years of age were accidentally poisoned by unspecified agents.

## DISCUSSION

Acute poisoning is a common medical emergency that contributes to morbidity and mortality worldwide.^2^ Studies from different parts of the world have reported various poisonous agents implicated in acute poisoning, including drugs, household chemicals and agricultural and industrial products.^5^ According to the 1995 estimate in the Botswana Economic Report, Botswana imports about 74% of the commodities used to sustain its fast growing economy in the industrial, healthcare and agricultural sectors. This has led to the introduction many hazardous poisonous agents that can accidentally or intentionally cause poisoning in both children and adults. A recent study at two hospitals in Francistown and Gaborone reported that there is a disparity with regard to the gender and age of the victims of acute poisoning. Although acute poisoning affected male and female patients equally, intentional poisoning was reported in 33.3% of the females versus 13.5% of the males. The majority of the victims were in the age category of 13 to 19 (20.7% versus 5.2%) for the females and in the 30-year-old group for the males (24.1% versus 10.3%). Moreover, poisoning by household chemicals affected mainly children below 12 years old, while poisoning by pharmaceuticals involved mainly teenagers.^6^

The findings of this study also confirm that there are differences with regard to the demographic status and residence of the victims, as well as with regard to the circumstances and outcomes of the poisoning incidents. For instance, the majority of poisoning incidents and eight of the nine deaths were recorded in the urban district of Gaborone than in the other districts. This could be explained by the great density of the population, and easy accessibility to products that are used for commercial, healthcare and industrial activities. In addition, the hospitals in the Gaborone district function as tertiary and referral hospitals that receive patients from other districts. In the rural districts, the majority of poisoning incidents were due to natural toxins from insects, scorpions and snakebites. This could be explained by the agrarian lifestyle of the population, and the fact that these districts are a suitable ecological habitat for insects and snakes.^7^

The findings of this study also show a difference in poisoning based on the age of the victims. The most vulnerable age group was that of children younger than six years (33.4%), followed by young adults aged 20 to 30 years. This finding is in accordance with other studies, which have indicated that children account for most poisoning cases, particularly children younger than five years.^8, 9^ Although it has been observed that children younger than five years have the highest hospitalisation rates due to poisoning, fatalities in this age group are usually uncommon.^10^ In this study, however, two deaths were reported in this age group. A significant drop in cases of poisoning was observed from children younger than five years (33%) to those aged six to 12 years (11%). However, poisoning incidents increased among young adults aged 20 to 30 years. Poisoning incidents among adults over 30 years were also higher than that of the age groups six to 12 years and 13 to 19 years. A similar trend was observed by Vale, who suggested that this phenomenon could be explained by the fact that incidents of poisoning decrease at the age of six to 12 as children develop less explorative behaviour.^11^

Regarding gender the results of this study showed that slightly more males than females were affected by poisoning, especially among children and adults over 30 years. These findings corroborate with those of previous work in this field.^12^ In contrast, there are a number of studies that have reported an overrepresentation of females in poisoning incidents among adolescents and adults over 30 years.^13^ Contrary to these findings, another study showed that nearly three quarters of poisoning admissions were for girls aged 15-17 years old, mainly intentional and due to ingestion of analgesics and psychotropic drugs.^14^

With regard to fatalities, an important finding from this study is that overdoses of medicine, both modern and traditional, resulted in the majority of deaths reported. This clearly points to the misuse of medicines for self-harm. This is of particular concern because the majority of deaths occurred among people older than 20 years. This trend could be an indication of underlining psychosocial problems and could also be related to attention-seeking behaviour and to peer pressure in the case of teenagers and young adults.^15^

With regard to the route of exposure, the results of this study show that the highest proportion (82.9%) of poisoning cases was through ingestion rather than other routes of exposure. Similar findings have been reported in other studies.^16, 17, 18^. The ingestion route was frequently used by adolescents and adults attempting self-harm, whereas such incidents among infants were accidental.

As for the toxic agents involved in these incidents, pharmaceuticals represented the majority of self-inflicted poisoning cases among adolescents and young adults that occurred in the three districts over the two-year period. In most cases of attempted suicide recorded, unknown drugs were ingested, followed by paracetamol (10.8%), potassium permanganate (6.4%), Amytriptyline (3.2%) and unspecified analgesics (3.2%). The use of such drugs could be attributed to the easy access to prescription drugs like painkillers and sedatives in the area of study. Paracetamol poisoning was also involved in accidental incidents among children younger than six years of age. This could be due to poor packaging or storage methods of such medicines in the homes, as well as a lack of vigilance among parents and caretakers. Poisoning by pharmaceutical was most common among young adults 20 to 30 years of age (38.9%), followed by adolescents 13 to 19 years of age (22.0%), where over 50% of the incidents were attempted suicide by drug overdose. There were more incidents among females than males (68.4% vs. 31.6%). Other retrospective studies have reported the misuse of over-the-counter drugs and prescription medicines.^19, 20^ This could be explained by possible poor vigilance over the accessibility of drugs either in the homes and/or at pharmaceutical outlets.

Increased availability coupled with poor storage measures explains why a considerable proportion of poisoning by household products was due to paraffin (62.8%), mainly among children younger than five years. Paraffin poisoning has also been reported to contribute significantly to morbidity among children in some South African rural areas.^14^ In addition; a few incidents of paraffin use by young adults 20 to 30 years of age in attempted suicides (14.0%) were recorded. Similar findings have been reported elsewhere. ^21, 22^ However, since poisoning by household products was not predominant in this study, this finding is contrary to studies conducted in developed countries, where household poisoning by chemicals is very common, especially among children who ingest cleansing agents like dishwasher soap and bleaches.^23^

With regard to natural toxins, children aged six to 12 years play in areas that are likely to be inhabited by insects and snakes, whereas adults encounter insects and snakes in their occupational environments, such as during farming and cattle rearing. Moreover, the common cases of accidental plant poisoning occurred in children younger than five years when they ingested the leaves of the elephant ear plant (*Colocasia*). This could be explained by the fact that this bulb is an ornamental plant commonly grown in home gardens. The bulb is soft and juicy, and the plant has green leaves that look attractive and edible to children who, at this stage, are unable to differentiate what is edible from what is not. A study by Vichova and Jahodor on plant poisoning in children reported a similar association between age and poisoning by plants.^24^

This study reported 14.4% confirmed cases of food poisoning. Food poisoning is one of the known major public health problems in Botswana, and affects mainly children under five years old. In this study, 32.9% of the victims were in this age group. This finding is consistent with a national report issued some years ago.^25^ There were also differences with regard to the distribution of food poisoning; although the majority of cases were recorded in Gaborone, followed by the South-East (37.6%) district, with only a few cases in the Kgatleng district (9.4%).

Although there were few cases of poisoning by traditional medicines, alcohol, agrochemicals and carbon monoxide, it is important to note that the majority of those poisoned by traditional remedies required hospitalisation, and two died. The use of traditional medicines and alcoholism are common in Botswana, and it is still unclear whether the frequencies of poisoning as found in this study reflect the real situation. However, this finding supports the study by But et al. (1996),^26^ who suggested that, in cases of poisoning in populations that have access to traditional medicines, the cause of morbidity or mortality may not be due to the traditional remedy but to other substances used in the preparation of the remedies. Because of the lack of toxicological screening procedures designed for the detection of traditional remedies in the hospital laboratories, there were no means to confirm suspicious cases besides the medical history related by the patients or relatives.

With regard to alcohol intoxication, it is well established that it is a major contributor to injuries, disabilities and deaths worldwide.^27^ However, this study found no reported fatalities associated with alcohol poisoning. The majority of cases were unintentional and among adults males aged 20 years old and above. As for agrochemical poisoning, most incidents were recorded in the Gaborone district and the agents were used in suicidal attempts. It is surprising that no cases of occupational exposure were recorded, as the use of agrochemicals in farming is common in the study areas. This finding is in contrast with other studies that reported higher rates of poisoning by agrochemicals in other countries.^28^

Carbon monoxide poisoning was rarely recorded over the two years and no fatality was due to it. The findings of this study are in agreement with those of a study conducted by Wilson et al., which showed that the elderly and children were at greatest risk from non-intentional carbon monoxide poisoning.^28^

This study suffered from some limitations. A major limitation was the fact that the data was based on hospital records of cases referred from clinics, resulting in the actual magnitude of the problem not being investigated fully. Further research involving data from sources such as clinics, coroners’ offices, forensic police, hospices and others would give a more representative picture of the magnitude of acute poisoning in Botswana. Another limitation stemming from the health system itself is the lack of toxicological screening facilities in most hospitals in Botswana, including the ones in this study. This lack could lead to misdiagnosis, as the health professionals can only depend on the patient history and have no means to identify the poisoning agents.

### Conclusion

In conclusion, it can be stated that acute poisoning involved mainly young children and resulted in an incidence rate of 4.7/1000, a case fatality rate of 3.8 /100, and 1.5% of deaths over the two-year period. There were differences based on age category, gender and residence of the victims, the types of toxic agents involved, as well as the circumstances and outcomes of the poisoning incidents. Given the fact that pharmaceuticals, natural toxins, household products and foods were the agents most commonly involved, targeted interventions should take into account these differences when addressing the problem of acute poisoning.
